# Full-field spectroscopic measurement of the X-ray beam from a multilayer monochromator using a hyperspectral X-ray camera

**DOI:** 10.1107/S1600577519015212

**Published:** 2020-01-01

**Authors:** Matthieu N. Boone, Frederic Van Assche, Sander Vanheule, Silvia Cipiccia, Hongchang Wang, Laszlo Vincze, Luc Van Hoorebeke

**Affiliations:** a Radiation Physics Research Group – UGCT, Department of Physics and Astronomy, Ghent University, Proeftuinstraat 86/N12, B-9000 Gent, Belgium; b Diamond Light Source, Diamond House, Harwell Science and Innovation Campus, Fermi Avenue, Didcot OX11 0DE, UK; c X-ray Microspectroscopy and Imaging Group, Department of Chemistry, Ghent University, Krijgslaan 281/S12, B-9000 Gent, Belgium

**Keywords:** multilayer monochromator, stripe artefact, full-field spectroscopy, hyperspectral X-ray detector, SLcam

## Abstract

Multilayer monochromator devices are commonly used at (imaging) beamlines of synchrotron facilities to shape the X-ray beam to relatively small bandwidth and high intensity. However, stripe artefacts are often observed and can deteriorate the image quality. Although the intensity distribution of these artefacts has been described in the literature, their spectral distribution is currently unknown. To assess the spatio-spectral properties of the monochromated X-ray beam, the direct beam has been measured for the first time using a hyperspectral X-ray detector. The results show a large number of spectral features with different spatial distributions for a [Ru, B_4_C] strip monochromator, associated primarily with the higher-order harmonics of the undulator and monochromator. It is found that their relative contributions are sufficiently low to avoid an influence on the imaging data. The [V, B_4_C] strip suppresses these high-order harmonics even more than the former, yet at the cost of reduced efficiency.

## Abstract 2

Full-field spectroscopic measurements of the X-ray beam from a multilayer monochromator are presented. With these measurements, the striping artefacts often found when using these devices can be fully characterized.

## 1. Introduction   

Multilayer monochromator devices (MLMs) are very popular devices at synchrotron facilities around the world, and imaging beamlines for high-resolution X-ray micro-computed tomography (microCT) in particular (Stampanoni *et al.*, 2007 ▸; Rack *et al.*, 2008 ▸, 2009 ▸; Wilde *et al.*, 2016 ▸; Weitkamp *et al.*, 2017 ▸). Their ability to shape the spectrum of X-ray beams to a bandwidth of several percent while maintaining a high flux (Görner *et al.*, 2001 ▸) makes them particularly useful for fast imaging applications (Rack *et al.*, 2010 ▸, 2011 ▸). Using adequate design parameters for the monochromators, the modified Bragg law followed by MLMs ensures a strong suppression of the second-order harmonic and usually also a mismatch between higher-order MLM harmonics and undulator harmonics, effectively resulting in very limited contamination with higher energies. However, the resulting beam often exhibits intensity fluctuations in space and time due to thermal instability of the optics (Titarenko *et al.*, 2010 ▸) and roughness of the surfaces (Rack *et al.*, 2010 ▸), which have a negative impact on the flat-field correction in tomographic reconstruction (Jailin *et al.*, 2017 ▸). The effects of these fluctuations are in many cases left unprocessed, taking advantage of the temporal averaging of X-ray microCT over the complete trajectory. In some cases, diffusers can be used to reduce these effects, albeit at the cost of reduced coherence (Rack *et al.*, 2010 ▸). Alternatively, specific post-processing algorithms are developed to reduce or compensate for these effects (Münch *et al.*, 2009 ▸; Van Nieuwenhove *et al.*, 2015 ▸). In all these methods, only the measured intensity is taken into account. This intensity corresponds to the integrated dose deposited in the scintillator material. Though the deposited dose depends on the spectral properties, the latter are not measured but rather assumed to be mono-energetic (after the MLM device). Nevertheless, a small spectral effect can have a large influence on the results, particularly when techniques such as *K*-edge subtraction (Roessl & Proksa, 2007 ▸; Mayo *et al.*, 2015 ▸; Thomlinson *et al.*, 2018 ▸; Porra *et al.*, 2018 ▸) or holotomography (Cloetens *et al.*, 1999 ▸) are applied.

## 2. Motivation   

**Figure 1:**
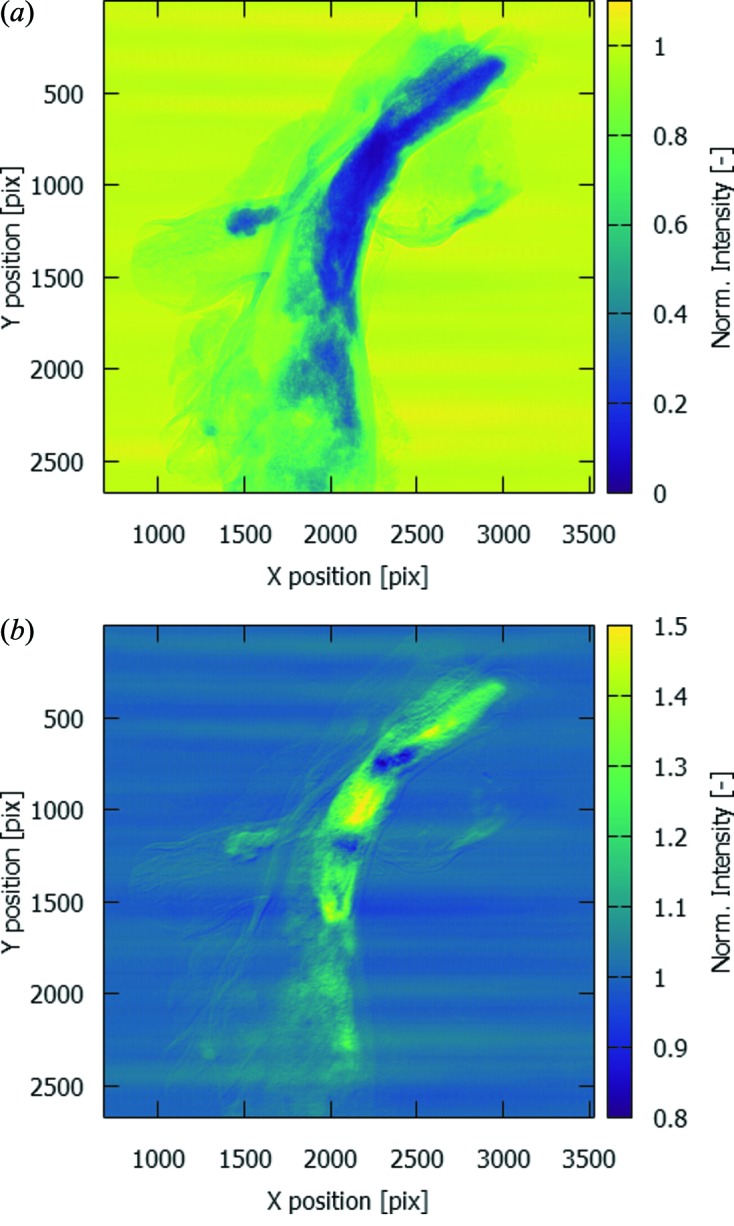
(*a*) Radiograph of a murine aortic arch with atherosclerotic plaque and gold nanoparticles; (*b*) division of the radiographs above and below the Au *L*_III_-edge.

**Figure 2:**
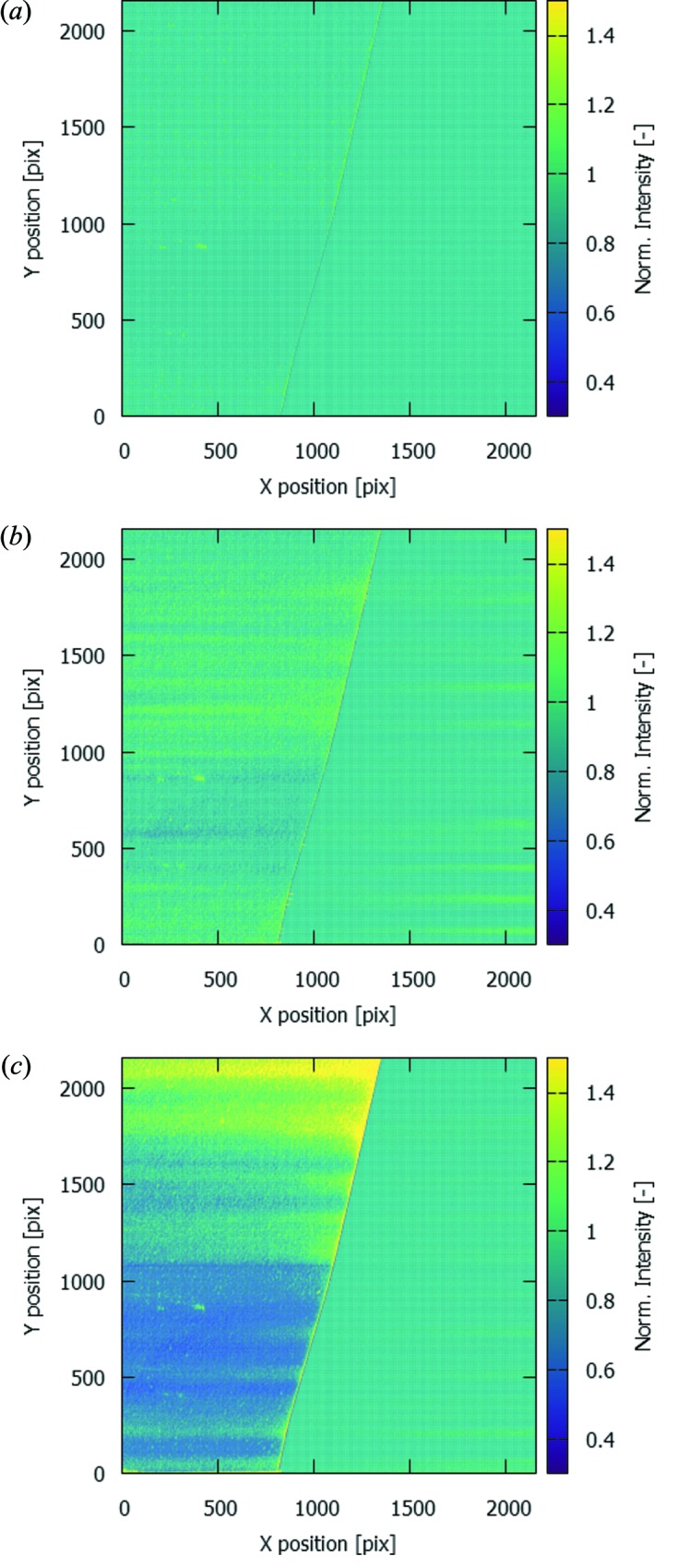
Normalized radiographic images of a 10 µm-thick gold foil (on the left in each image) using (*a*) pink beam, (*b*) MLM monochromator below the Au *L*_III_-edge, and (*c*) MLM monochromator above the Au *L*_III_-edge. The normalization process is elaborated on in the text.

In a recent beam time at Diamond Light Source I13-2, edge subtraction around the Au *L*_III_-edge was applied to retrieve the localization of gold nanoparticles in murine aortic arches with atherosclerotic plaque (Cormode *et al.*, 2010 ▸; Chhour *et al.*, 2016 ▸). Analysis of these data revealed a suspiciously striped spatial distribution of the gold signal, showing large areas of gold signal without correlation with morphological features in the aortic arch (Fig. 1 ▸). Repeated tests using a thin gold foil (10 µm thickness) suggested a similar issue, yet these tests were not conclusive, as the intensity fluctuations make the normalization unreliable. To compensate for these fluctuations, additional normalization is performed for each detector row individually by using a small region next to the foil edge, measuring the open-beam signal at this row. The foil edge is determined as the maximum of the first derivative of the horizontal line profile in the proximity of the edge as visually determined. To avoid physical edge effects of the foil, these small regions were taken at 10 pixels distance from the determined edge. The width of these regions was 200 pixels (out of a total 2560 pixels), which was found to be a good trade-off to obtain sufficient statistics (reducing noise) while being sufficiently small to limit the influence of effects from the horizontal variation of the streaks. To improve the visibility of the figure, the region covered by the foil is additionally normalized to the average intensity over the full foil in the normalized image. In Fig. 2 ▸, it can be observed that the vertical streaks are clearly visible in the measurement above the Au *L*-edge [Fig. 2(*c*) ▸], whereas they are much less pronounced in the measurement just below the Au *L*-edge [Fig. 2(*b*) ▸]. As they are not visible in the pink beam measurement [Fig. 2(*a*) ▸], they cannot be attributed to thickness changes of the gold foil itself.

In this paper we investigate for the first time the spatio-spectral distribution of the X-ray beam shaped by a multilayer monochromator to experimentally evaluate the suppression of higher-order harmonics and elucidate the possible impact on energy-sensitive experiments such as edge subtraction.

## 3. Materials and methods   

### 3.1. Diamond Light Source I13-2 beamline   

The Diamond Light Source beamline I13 for imaging and coherence applications is dedicated to hard X-ray imaging on the micro and nano length scale. In one of the long straight sections of the 3 GeV storage ring, two insertion devices (IDs) are placed to enable independent operation of two stations, I13-1 and I13-2, at a distance of more than 200 m from the ID (Rau *et al.*, 2011 ▸). In this study, the imaging branch I13-2 is used, which is based on a 2 m-long U22 undulator device. The so-called mini-β layout allows for operation at very small undulator gaps down to 5 mm for improved brilliance, particularly at higher energies (Rau *et al.*, 2011 ▸). The front-end slits are positioned 18 m downstream from the ID, and the deflecting mirror at 32 m downstream. Without using focusing optics, the beam size in the experimental hutch is about 16 mm × 9 mm.

The I13-2 branchline has two different monochromator devices available. The MLM, positioned directly downstream of the deflecting mirror, is used for high-flux applications where monochromatic radiation is required but spectral bandwidth is of less importance. It is equipped with three different options for the strip material: a [Ru,B_4_C] strip (100 layers at 4.588 nm spacing, hereon denoted MLM-Ru), a [V,B_4_C] strip (400 layers at 2.505 nm spacing, hereon denoted MLM-V) and a [Mo,B_4_C] strip (400 layers at 2.003 nm spacing, not used for these experiments), which allow for a spectral bandwidth of approximately 10^−2^. For all three strip materials, a γ ratio (*i.e.* the ratio of the thickness of B_4_C over the periodic thickness) of 0.57 is chosen in the beamline design, optimizing the reflectivity rather than suppression of higher-order harmonics. In most cases, these higher-order harmonics are further suppressed using suitable mirrors. In the experiments mentioned earlier, a Si strip mirror is used, which has a reflectivity of the order of 2 × 10^−2^ at 24.0 keV, the energy of the second harmonic. For applications requiring high temporal coherence (bandwidth of the order of 10^−4^), a Si[111] double-crystal monochromator (hereon denoted DCM) is available, which is located just upstream of the experimental hutch.

The I13-2 imaging station is dedicated to micro- and nano-imaging in the range 8–35 keV, with resolutions from the micrometre range to better than 100 nm (Vila-Comamala *et al.*, 2016 ▸). The beamline is equipped with different detector systems with a modular and interchangeable composition of scintillator screen, microscope objective and CCD or sCMOS sensor (De Fanis *et al.*, 2013 ▸) for different acquisition protocols. In this work, the standard available detector system was only used for beam alignment [notably when compensating for the vertical offset introduced by the DCM (Pešić *et al.*, 2013 ▸)] and fast inspection of new settings, while a hyperspectral X-ray camera was used for full-field spectroscopic measurements.

### 3.2. Hyperspectral X-ray camera   

The spatial and spectral distribution of the beam was measured using a colour X-ray camera or SLcam hyper­spectral detector (Scharf *et al.*, 2011 ▸). This detector, based on a 450 µm-thick Si pnCCD sensor (pnSensor, Munich, Germany), has an active area of 264 × 264 pixels of size 48 µm × 48 µm which is read out at approximately 400 Hz. In the sensor, each detected X-ray photon induces a charge cloud, typically spanning multiple pixels (Kimmel, 2008 ▸). When the incident photon flux is sufficiently low, the charge clouds are observed separately in the read out frames, allowing for accurate reconstruction of the total deposited charge and location of the interaction (Kimmel *et al.*, 2006 ▸; Ordavo *et al.*, 2011 ▸; Cartier *et al.*, 2016 ▸). Its high-end readout electronics are interfaced with a new and in-house-developed control and processing software chain (Van Assche *et al.*, 2018*b* ▸), resulting in an energy resolution of approximately 150 eV (at Mn *K*α) and a spatial resolution better than the pixel size.

### 3.3. Constraints on the measurement conditions   

The drawback of this detector system is its limited count rate capability (Boone, 2013 ▸; Boone *et al.*, 2014 ▸). This is primarily induced by the requirement of having sparse frames, *i.e.* frames in which all the detected charge clusters can be observed individually and separately. Overlapping clusters result in inaccurate position determination and pulse pile-up. A non-linear response is found even at very low count rates, and starts to dominate the image at approximately 5–10 counts pixel^−1^ s^−1^, or approximately 2500 counts s^−1^ mm^−2^.

To effectively measure the direct X-ray beam using the hyperspectral X-ray camera, it is key to reduce the flux in the beam. In this work, two approaches are used. A straightforward means to achieve this is to add a large amount of beam filtering. Though this effectively reduces the flux, it drastically changes the spectrum of the beam. As a second means, the primary slits after the undulator source are closed as much as possible, yet this also alters the properties of the beam. High-repetition-rate mechanical choppers (Osawa *et al.*, 2017 ▸) are not considered for this work as they do not offer sufficient flexibility in attenuation strength of the flux, which needed to span several orders of magnitude for this work. An overview of the beam filtration used in this work as well as the undulator gap, the monochromator angle and the mirror material are given in Table 1 ▸.

Using beam filtration, beam attenuation is spectrally very inhomogeneous, strongly reducing the low-energy radiation while leaving high-energy X-rays relatively undisturbed. This is a very undesired effect, particularly considering the low sensitivity of the SLcam sensor to high-energy radiation and increased chance of radiation damage inside the readout electronics. As a result, the practical range for the main investigated energy of the beam (*i.e.* the fifth undulator harmonic coinciding with the fundamental MLM reflectivity) was limited to between 11 keV and 18 keV. For lower fundamental energies, the primary beam was fully absorbed by the attenuators required to sufficiently reduce the intensity of the high-order harmonics, while for higher energies under investigation the energy of the higher-order harmonics became too high for the sensitive camera system.

### 3.4. Data analysis   

During the experiment, raw frames from the SLcam were stored for off-line analysis. This approach allowed for further improvement of the energy resolution of the SLcam camera based on the experimental data using the DCM, exploiting the extremely small energy bandwidth of the X-ray beam. A custom calibration, thresholding and cluster analysis method is used to segment the separate charge clusters and assess their properties (Van Assche *et al.*, 2018*a* ▸). This also allows to discriminate between small clusters and large clusters, of which the latter are more likely to be the cause of pulse pileup. For each cluster size (in number of pixels above threshold), a separate datacube is generated, each containing 4000 images of 264 × 264 pixels, equally distributed between 0 keV and 40 keV hence at an effective bin width of approximately 10 eV. Fine-tuning of the offset and gain calibration is performed on the fluorescent radiation peaks of Fe, Cu and Mo (see Table 2 ▸).

The datacubes are analyzed using custom analysis software. The analysis workflow is based on the sum spectrum of all pixels in order to identify the different spectral features. Regions of interest (ROIs) in the spectrum are selected manually to isolate single peaks. Where possible (*i.e.* when the peak is sufficiently isolated), a background correction is performed based on linear interpolation between the intensity at the beginning and at the end of the spectral ROI. For partially overlapping peaks, a manual background correction is performed. Given the nature of these data, the background is relatively low and has limited influence on the final results. A Gaussian function is fitted to the corrected peaks, from which the range μ ± 3σ is used as updated ROI, where μ and σ are the mean and the standard deviation of the Gaussian fit, respectively.

The spatial intensity distribution for each peak in the spectrum is determined by summing the spatial distributions of the energy bins within this updated ROI. Where applicable, the intensity is corrected for the beam filtration and detector efficiency by attributing the whole peak to its central energy.

The same updated spectral ROIs are used to determine the temporal stability of the spectrum. Within the spectral ROI of the main peak under investigation, the mean energy and standard deviation are determined within a small temporal interval. As there are only a few counts per raw frame, the spatial information is discarded for this aspect. The analysis is performed on the first 400000 frames from the stored raw data for both experimental settings 1 and 3. To reduce the influence of the low statistics per sample point, the analysis was performed on both the raw frame series at approximately 400 Hz and a rebinned series at approximately 40 Hz. To evaluate these data, the Fourier power spectrum was determined for each dataset after subtraction of the mean value and compared with simulated zero-centred Gaussian noise with the same standard deviation.

## 4. Results   

### 4.1. Identification of spectral features   

**Figure 3:**
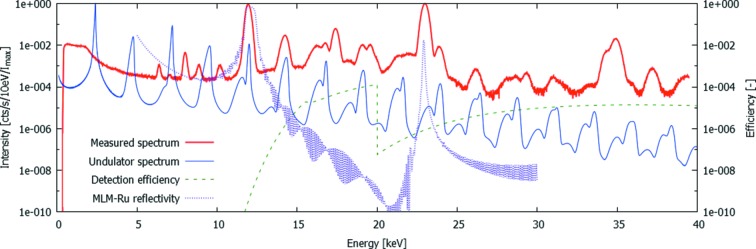
Measured spectrum using ES1 (full line). Additional plots show the simulated undulator spectrum (Tanaka & Kitamura, 2001 ▸), the MLM efficiency (reflectivity) as calculated from Kohn (1995 ▸) and the detection efficiency, dominated by the high amount of beam filtering at low energies and the low interaction chance in the sensor and the low reflectivity of the Si mirror at high energies.

A typical sum spectrum from these experiments is shown in Fig. 3 ▸, extracted from the acquisition using ES1 (Table 1 ▸). The spectrum shows a very large number of features, which can be divided into features present in the primary beam and features originating from physical effects inside the SLcam system. This should particularly be kept in mind while correcting for the energy-dependent attenuation caused by the beam filtration and the detection probability in the 450 µm-thick sensor. The convolution of these two effects is plotted as the detection efficiency in Fig. 3 ▸. It should be noted that this efficiency is only applicable to the primary beam features, and not for the internal effects such as the low-energy tail and fluorescent peaks. In Table 2 ▸, all identified peaks are listed and explained, indicating their origin, their relative intensity *I* per second per pixel or where applicable the intensity *I*_cor_ corrected for the detection efficiency.

It is clear from this table that the detected beam contains a large number of undesired peaks in a quasi-monochromatic X-ray beam, yet at a very low amplitude. Despite the MLM, both the sixth and seventh harmonic are clearly visible in the spectrum due to the spectral effects of the strong filtering. Above the seventh harmonic, most peaks are successfully filtered out by the combination of the MLM and the Si strip mirror. Furthermore, it is noteworthy that the harmonic of the MLM does not correspond to exactly twice the fundamental energies. This can be precisely calculated (from http://henke.lbl.gov/optical_constants/) (Kohn, 1995 ▸) and is particularly interesting in this case as it allows to discriminate the real contribution of the primary beam from the sum peak caused by two events which have been detected near each other during the same readout frame, causing the detected clusters to overlap. For the evaluation of this pileup, the contribution of the tenth undulator harmonic is assumed to be negligible as predicted by simulations.

**Figure 4:**
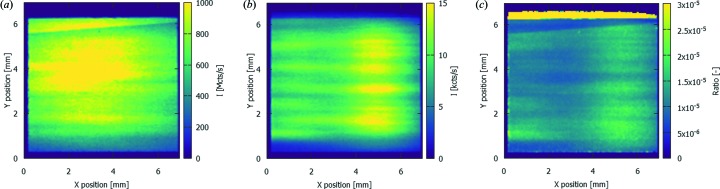
Spatial distribution of the incident count rate for (*a*) the MLM-Ru fundamental energy at 12.0 keV and (*b*) the MLM-Ru harmonic energy at 23.1 keV, measured using ES1. Panel (*c*) shows the ratio between both intensities. The count rates in this figure are corrected for the attenuation of the beam by the filters, the detection efficiency of the 450 µm-thick sensor of the SLcam and the reflectivity of the Si strip mirror.

The spectrum shown in Fig. 3 ▸ was summed over the full illuminated area. However, in the scope of this work it is most important to assess the spatial distribution of this spectrum. Figs. 4(*a*) and 4(*b*) ▸ show the intensity distribution of the MLM-Ru fundamental peak at 12.0 keV and the MLM-Ru harmonic at 23.1 keV, respectively. This distribution is clearly different, and Fig. 4(*c*) ▸ shows the ratio between the intensity of these two as a function of position in the beam. It should be noted that the intensities plotted in Fig. 4 ▸ are already corrected for the attenuation of the incident beam and the detection efficiency, hence Fig. 4(*c*) ▸ is also an indication for the relative intensities of these two peaks in imaging experiments at this beamline. From this result, it is clear that, without additional filtering, the intensity of the harmonic is of the order of magnitude of 10^−5^ and can thus be considered negligible. It is important to note that approximately two orders of magnitude can be attributed to the Si strip mirror.

### 4.2. MLM strip material and spacing   

**Figure 5:**
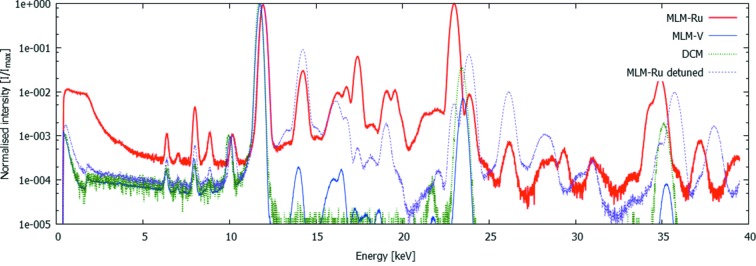
Measured spectra using different experimental settings. The spectra are normalized to their maxima. It is important to note that the filtering is different for different measurements, hence no comparison on the amplitudes or ratios of the different peaks should be made.

Two different available MLMs were evaluated and compared. They have a different strip material (V and Ru) and a different spacing (4.588 nm and 2.505 nm, respectively). The data were acquired using ES1 and ES3, respectively. The spectra of both are shown in Fig. 5 ▸. It is important to note that the beam filtration was not identical for both experiments, hence the intensities and ratios between different peaks cannot be directly compared in this figure. Nevertheless, it is clear that the contribution of higher-order harmonics is further reduced.

**Figure 6:**
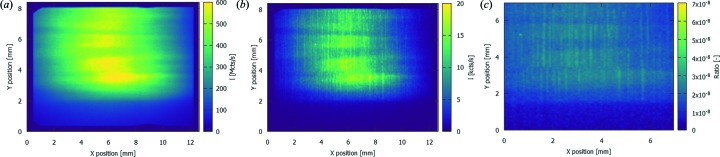
Spatial distribution of the incident count rate using the MLM-V for (*a*) the fundamental energy 12.0 keV and (*b*) the peak at 23.7 keV, measured using ES3. Panel (*c*) shows the ratio between both intensities. The count rates in this figure are corrected for the attenuation of the beam by the filters, the detection efficiency of the 450 µm-thick sensor of the SLcam and the reflectivity of the Si strip mirror.

**Figure 7:**
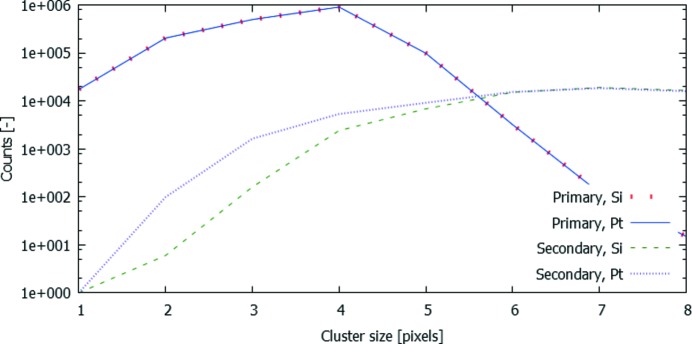
Number of counts per event size for the fundamental peak (denoted ‘Primary’) and the peak containing both pileup events and high-order harmonics (denoted ‘Secondary’) using both a Si strip mirror and a Pt strip mirror.

Most notably, the harmonic of the MLM-V and the pulse pileup peak of the fundamental coincide in this case. This makes it impossible to discriminate between the two effects. However, Fig. 6 ▸ shows that the pattern of the fundamental and the peak at 23.7 keV are very similar, and the ratio between both [Fig. 6(*c*) ▸] hints towards a very strong contribution of pulse pileup rather than the MLM-V harmonic, despite the large amount of filtering. This is confirmed by the absence of Mo fluorescence, as there is only a negligible amount of radiation above the Mo *K*-edge. When a Pt mirror is used instead of the Si mirror, the small amount of radiation in the MLM-V harmonic is maintained better and the Mo *K*α peak becomes visible. Additionally, the contribution of the MLM-V harmonic can be investigated by analyzing the cluster size distribution in ES6 and ES7, using the Si and Pt strip mirror, respectively. Indeed, as pulse pileup events are very likely to be relatively large events, it is expected that more small events are visible in ES6. This is shown in Fig. 7 ▸, where the number of events is plotted as a function of the event size for both the 11.7 keV and the 23.4 keV peak for both strip mirrors. As expected, the amount of small events in the 23.4 keV peak is much higher when using a Pt strip mirror due to the contribution of the MLM-V harmonic. The curves for the 11.7 keV peak, on the other hand, coincide almost perfectly. Unfortunately, due to the low count rates, no spatial information can be deduced from this comparison.

### 4.3. Temporal effects   

The Fourier power spectrum of the mean energy function over time of the first 400000 frames of ES1 (ES3) has a standard deviation of 33.3 eV (19.5 eV) for the unbinned data and a standard deviation of 10.1 eV (6.0 eV) for the data using temporal binning. Despite this extremely high accuracy, the analysis reveals no additional modes with a higher amplitude than what can be found in simulated series. As such, it is highly unlikely that there is any temporal effect in the mean energy of each peak at a temporal resolution below 200 Hz.

### 4.4. Detuning of second crystal   

**Figure 8:**
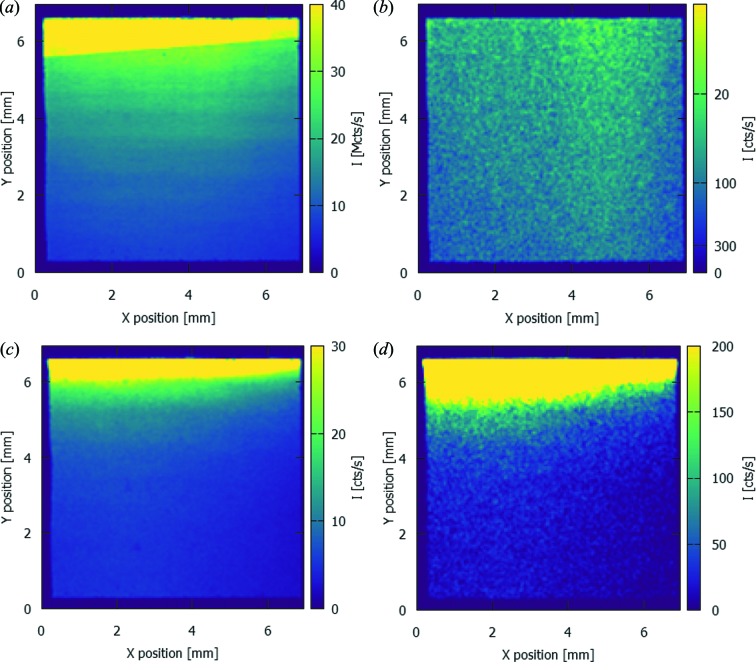
Spatial distribution of the incident count rate for the fundamental energy [(*a*) and (*c*)] and the first harmonic [(*b*) and (*d*)] using the detuned second crystal of the MLM-Ru [ES2, (*a*) and (*b*)] and MLM-V [ES4; (*c*) and (*d*)]. These count rates are corrected for the attenuation of the beam by the filters and the detection efficiency of the 450 µm-thick sensor of the SLcam. The high-intensity regions at the top of the image are due to incomplete filtering of the field-of-view.

A well known means to suppress the spatial intensity variation of the beam is to detune the second crystal of the MLM. This is evaluated with ES2 and ES4. As observed in Fig. 8 ▸, the spatial intensity modulations have indeed disappeared, both in the fundamental and the harmonic peak. However, particularly for the MLM-Ru, the ratio between the intensity of the fundamental energy peak and the first harmonic has remained similar to the results of the fully optimized setting.

## 5. Discussion   

**Figure 9:**
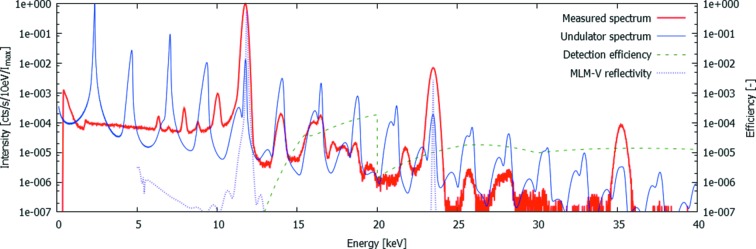
Measured spectrum using ES3 (full line). Additional plots show the simulated undulator spectrum (Tanaka & Kitamura, 2001 ▸), the MLM efficiency (reflectivity) as calculated from Kohn (1995 ▸) and the total detection efficiency, dominated by the high amount of beam filtering at low energies and the low interaction chance in the sensor and the low reflectivity of the Si mirror at high energies.

The absence of a harmonic when using the MLM-V in combination with a Si strip mirror (Fig. 9 ▸) corresponds to the calculated reflectivity of the MLM-V as compared with the MLM-Ru, which is shown in Fig. 9 ▸. Indeed, the reflectivity of the harmonic is more than a magnitude lower for the MLM-V than for the MLM-Ru. At the same time, also the reflectivity of the fundamental energy is lower, resulting in a decreased efficiency of the monochromator. The choice of the MLM strip material therefore remains a trade-off between beam quality and X-ray flux.

Though detuning of the second crystal of the MLM has been shown to be a good means to eliminate the intensity modulations in the beam (Fig. 8 ▸), it is important to compare this result with the performance of a DCM, which is known for high-quality beams (small spectral bandwidth, homogeneous beam). For a proper comparison, the measurement of ES4 is included in Fig. 5 ▸. In the full-field measurement of ES4, no intensity modulations are observed (not shown). The two figures of merit in this comparison are the spectral bandwidth of the (fundamental) peak and the flux at the detector. On the first aspect, there is no substantial influence of the detuning and therefore the DCM still outperforms the MLMs. On the second aspect, however, despite the reduction in efficiency due to the detuning, the MLMs are found to provide higher fluxes in the fundamental peak than the DCM: 9.2 × 10^9^ photons s^−1^ mm^−2^ and 4.60 × 10^9^ photons s^−1^ mm^−2^ for the MLM-Ru and the MLM-V, respectively, versus 2.82 × 10^9^ photons s^−1^ mm^−2^ for the DCM. For applications where beam homogeneity and flux are important and spectral bandwidth is less critical (but too important to rule out pink beam mode), *e.g.* high-speed radiographic imaging, this technique is therefore still a valid alternative.

A noteworthy effect visible in Fig. 8 ▸ is the region with high intensity at the top of the image. This is an artefact due to incomplete coverage of the beam by one of the filters. This region is clearly visible at the fundamental energy for both strip materials, yet it is barely visible at the harmonic of the MLM-Ru. For the 24.0 keV peak using the MLM-V, this region is clearly visible, again suggesting a strong contribution of pulse pileup rather than observation of the MLM harmonic.

An important limitation of the discussed measurements is the low temporal resolution for the resulting full-field images, as the complete exposure time was typically 30 min. Although some information at higher temporal resolution is available due to the 400 Hz frame rate (see Section 4.3), the number of photons detected per frame is very low (approximately 1 photon per 10 pixels). Therefore, retrieving direct information from these datasets is like looking for a needle in a haystack. Alternative setups using a beam splitter to simultaneously measure the intensity distribution of the beam at high speed with sufficient photon statistics (using a conventional camera) and simultaneously measure the spectroscopic distribution of a small portion of the beam (using a hyperspectral X-ray camera) may provide this information.

## 6. Conclusion   

This paper presents the spatio-spectral distribution of a synchrotron beam after a multilayer monochromator. For the first time, the spectral characteristics of the striping pattern, typical for MLMs, is characterized. The pattern is shown to be different for different energies present in the beam (fundamental and higher-order harmonics), yet most are several orders of magnitude smaller than the primary energy by design. Furthermore, this contribution is further reduced with a well chosen reflective mirror further downstream of the beam. Detuning of the second crystal is an effective solution to eliminate the intensity modulations in the beam, but reduces the intensity of the beam drastically. However, still a higher flux is maintained as compared with using a double-crystal monochromator.

## Figures and Tables

**Table 1:** Experimental settings (ES) of different experiments In addition to the stated filtering, 1.34 mm of pyrolitic graphite was used for all experiments.

ES	Monochromator	ID gap [mm]	θ_B_ [°]	Mirror	Al [mm]	Mo [µm]	Ag [µm]
1	MLM-Ru	8.13	0.6889	Si	3.7	114	0
2	MLM-Ru†	8.13	0.6889	Si	2.4	114	0
3	MLM-V	7.96	1.2120	Si	3.2	72	35
4	MLM-V†	7.96	1.2120	Si	2.4	114	0
5	DCM	7.96	9.645	Si	2.4	114	0
6	MLM-V‡	7.96	1.2120	Si	3.2	0	0
7	MLM-V‡	7.96	1.2120	Pt	3.2	0	0

† Detuned measurements.

‡ Measurements made with mall primary slit openings to reduce the X-ray flux.

**Table 2:** Spectral features present in a measured spectrum as shown in Fig. 3 ▸ The corrected intensity *I*_cor_ for the sum peak (denoted with an asterisk) indicates the expected original number of 11.98 keV photons, neglecting the amplitude of the tenth undulator harmonic.

Energy [keV]	Origin	*I* (*I*_cor_) [counts s^−1^ pixel^−1^]	Explanation
6.41 (7.06	Detector	2.79 × 10^−4^ (1.29 × 10^−4^)	Fe fluorescence from housing
8.04 (8.90)	Detector	1.03 × 10^−3^ (3.63 × 10^−4^)	Cu fluorescence from housing
10.22	Beam	1.14 × 10^6^	Fundamental energy escape peak
11.98	Beam	7.24 × 10^8^	Fundamental energy (5th undulator harmonic)
14.29	Beam	7.43 × 10^3^	6th undulator harmonic
16.34 (16.83)	Beam	1.02 × 10^2^ (3.44 × 10^1^)	7th undulator harmonic
17.47 (19.64)	Detector	2.36 × 10^−2^ (4.78 × 10^−3^)	Mo fluorescence from radiation shield
19.17	Beam	3.67	8th undulator harmonic
23.09	Beam	9.10 × 10^3^	Monochromator harmonic
23.98	Hybrid	2.05 × 10^7^*	Sum peak of fundamental; 10th undulator harmonic
26.28	Beam	1.09	11th undulator harmonic
